# Supplementary material for: Formulation and in-vitro functional evaluation of a Bacillus-based multi-strain probiotic consortium relevant to protein-energy malnutrition
Source: PLoS One. 2026 Mar 24;21(3):e0345821. doi: 10.1371/journal.pone.0345821 (PMC13012502; doi:10.1371/journal.pone.0345821)
Supplement: S1 Table — (DOCX) [file pone.0345821.s001.docx]

**S1 Table:** Gram staining, negative staining, catalase and oxidase test results of selected 23 isolates with standard strains *Lactiplantibacillus plantarum* NCDC 347, *Lacticaseibacillus rhamnosus* NDRI 184

| **Sr. no.** | **Isolates no.** | **Source** | **Colony morphology** | **Gram staining** | **Negative staining** | **Oxidase test** | **Catalase test** |
| --- | --- | --- | --- | --- | --- | --- | --- |
|  | *Lactiplantibacillus plantarum* NCDC 347 |  | Round, white, creamy, entire margined, small, opaque | +ve | Long rod | - | - |
|  | *Lacticaseibacillus rhamnosus* NDRI 184 |  | Round, large, creamy, white colonies | +ve | Long rod | - | - |
| 1 | PIG5CI | Goat Milk | Yellowish, Small, irregular, stick, entire, Shiny, opaque, Sticky | +ve | Long rod | - | - |
| 2 | PIG3IR | Goat Milk | White, Big, irregular, dry, convex, Flat. | +ve | Short rod | - | - |
| 3 | PIG6IR | Goat Milk | White, Big, irregular, dry, convex, Flat | +ve | Short rod | - | - |
| 4 | PIB13MR | Buttermilk | Maroon, irregular, flat, dry | +ve | rod | - | - |
| 5 | PIB14TR | Buttermilk | Spread like, thread, round | +ve | rod | - | - |
| 6 | PIB12FI | Buttermilk | Flat, cream, irregular | +ve | rod | - | - |
| 7 | PIB12RB | Buttermilk | Rice like colony | +ve | rod | - | - |
| 8 | PIM10FI | Milk | Flat, cream, irregular, dry | +ve | rod | - | - |
| 9 | PIY1RC | Yogurt | Round, white, creamy, entire margined, small | +ve | rod | - | - |
| 10 | PIC20SC | Curd | Small, creamy, creasy, round, raised | +ve | rod | - | - |
| 11 | PIC20SY | Curd | Small, yellow, dry, round, raised | +ve | rod | - | - |
| 12 | PIC23R | Curd | Small, round, white, flat, dry | +ve | rod | - | - |
| 13 | PIC22IF | Curd | Irregular, entire, flat, creamy, sticky | +ve | rod | - | - |
| 14 | PIC22RI | Curd | Off-white, small, round, irregular | +ve | rod | - | - |
| 15 | PIM8CR | Milk | Creasy, irregular, sticky, yellow | +ve | rod | - | - |
| 16 | PIC5CR | Curd | Creasy, irregular, sticky, yellow | +ve | rod | - | - |
| 17 | PIM9FI | Milk | Flat, cream, irregular | +ve | rod | - | - |
| 18 | PIM9CR | Milk | Creasy, irregular, sticky, yellow | +ve | rod | - | - |
| 19 | PIB9SR | Buttermilk | Small, round, white, flat, dry | +ve | rod | - | - |
| 20 | PIB10CR | Buttermilk | Creasy, irregular, sticky, yellow | +ve | rod | - | - |
| 21 | PIB10MR | Buttermilk | Maroon, irregular, flat, dry | +ve | rod | - | - |
| 22 | PIB9MR | Buttermilk | Maroon, irregular, flat, dry | +ve | rod | - | - |
| 23 | PIB9CR | Buttermilk | Creasy, irregular, sticky, yellow | +ve | rod | - | - |

**All experiments were performed in triplicates; Data represented as Mean±SD**

**Foot note: “-“ represent negative**
