# Supplementary material for: Formulation and in-vitro functional evaluation of a Bacillus-based multi-strain probiotic consortium relevant to protein-energy malnutrition
Source: PLoS One. 2026 Mar 24;21(3):e0345821. doi: 10.1371/journal.pone.0345821 (PMC13012502; doi:10.1371/journal.pone.0345821)
Supplement: S2 Table — (DOCX) [file pone.0345821.s002.docx]

**S2 Table.** Secondary screening results of selected 23 isolates with standard strains *Lactiplantibacillus plantarum* NCDC 347, *Lacticaseibacillus rhamnosus* NDRI 184

| **Sr. no.** | **Isolates no.** | **Acid tolerance (pH)** | **Bile tolerance (%)** | **Cell autoaggregation (%)** | **Cell surface hydrophobicity (Xylene) (%)** | **Cell surface hydrophobicity (Toluene) (%)** |
| --- | --- | --- | --- | --- | --- | --- |
|  | *Lactiplantibacillus plantarum* NCDC 347 | 1.5 | 2 | 98.7±1.0 | 64.1±2.4 | 42.9±2.2 |
|  | *Lacticaseibacillus rhamnosus* NDRI 184 | 1.5 | 2 | 98.9±0.2 | 70.1±5.1 | 46.5±4.0 |
| 1 | PIG5CI | 2 | 2 | 86.1±0.2 | 62.1±1.9 | 42.3±8.5 |
| 2 | PIG3IR | 1.5 | 2 | 86.9±0.8 | 64.2±1.6 | 73.6±4.9 |
| 3 | PIG6IR | 2 | 2 | 87.5±0.5 | 79.2±3.5 | 66.5±8.6 |
| 4 | PIB13MR | 1.5 | 2 | 87.3±0.5 | 71.2±0.4 | 46.5±3.6 |
| 5 | PIB14TR | 3 | 2 | 88.7±0.9 | 70.9±1.4 | 67.9±1.6 |
| 6 | PIB12FI | 1.5 | 2 | 81.4±1.2 | 51.6±1.4 | 65.9±2.1 |
| 7 | PIB12RB | 1.5 | 2 | 89.7±0.5 | 61.2±2.6 | 58.0±2.0 |
| 8 | PIM10FI | 2 | 2 | 87.4±2.0 | 91.6±0.9 | 84.7±0.6 |
| 9 | PIY1RC | 1.5 | 2 | 86.2±0.9 | 76.3±2.8 | 92.9±0.3 |
| 10 | PIC20SC | 1.5 | 2 | 98.6±0.1 | 80.9±5.7 | 95.1±0.1 |
| 11 | PIC20SY | 2 | 2 | 86.6±0.3 | 77.4±0.6 | 93.7±0.6 |
| 12 | PIC23R | 1.5 | 2 | 99.4±0.0 | 95.8±0.1 | 95.2±0.4 |
| 13 | PIC22IF | 2 | 2 | 98.9±0.1 | 84.9±0.2 | 90.0±1.2 |
| 14 | PIC22RI | 1.5 | 2 | 98.3±0.0 | 90.7±1.6 | 96.4±0.1 |
| 15 | PIM8CR | 1.5 | 2 | 83.6±1.3 | 84.6±1.4 | 78.0±2.0 |
| 16 | PIC5CR | 1.5 | 2 | 88.0±0.4 | 90.8±0.7 | 88.8±1.4 |
| 17 | PIM9FI | 1.5 | 2 | 88.7±2.0 | 89.8±1.9 | 89.0±1.7 |
| 18 | PIM9CR | 2 | 2 | 88.1±2.1 | 89.2±0.8 | 90.4±0.4 |
| 19 | PIB9SR | 2 | 2 | 86.4±0.3 | 78.3±2.5 | 90.2±0.3 |
| 20 | PIB10CR | 1.5 | 2 | 84.7±2.8 | 84.9±2.0 | 76.4±4.3 |
| 21 | PIB10MR | 2 | 2 | 88.0±1.3 | 88.6±3.8 | 88.2±2.1 |
| 22 | PIB9MR | 2 | 2 | 81.1±2.9 | 88.8±0.7 | 75.3±3.4 |
| 23 | PIB9CR | 2 | 2 | 81.3±1.4 | 91.1±0.8 | 88.0±4.1 |

**All experiments were performed in triplicates; Data represented as Mean±SD**
