# Supplementary material for: Formulation and in-vitro functional evaluation of a Bacillus-based multi-strain probiotic consortium relevant to protein-energy malnutrition
Source: PLoS One. 2026 Mar 24;21(3):e0345821. doi: 10.1371/journal.pone.0345821 (PMC13012502; doi:10.1371/journal.pone.0345821)
Supplement: S3 Table — (DOCX) [file pone.0345821.s003.docx]

| **Sr. no.** | **Isolates no.** | **Reducing power (%) of FRAP** | **% reduction of ABTS** | **% reduction of DPPH** |
| --- | --- | --- | --- | --- |
|  | *Lactiplantibacillus plantarum* NCDC 347 | 65.40±0.41 | 85.16±0.92 | 39.69±4.63 |
|  | *Lacticaseibacillus rhamnosus* NDRI 184 | 78.18±0.08 | 85.61±0.45 | 39.56±0.23 |
| 1 | PIG5CI | 80.93±0.12 | 78.33±0.52 | 16.40±1.34 |
| 2 | PIG3IR | 83.66±0.05 | 78.75±0.22 | 9.28±1.70 |
| 3 | PIG6IR | 75.96±0.18 | 55.72±0.92 | 29.95±0.40 |
| 4 | PIB13MR | 79.67±0.07 | 76.02±0.45 | 9.10±2.25 |
| 5 | PIB14TR | 75.78±0.05 | 42.09±0.94 | 20.66±0.73 |
| 6 | PIB12FI | 78.80±0.08 | 69.80±0.68 | 4.40±1.39 |
| 7 | PIB12RB | 80.26±0.05 | 83.45±0.39 | 26.46±1.75 |
| 8 | PIM10FI | 72.20±0.08 | 90.59±0.52 | 10.04±0.40 |
| 9 | PIY1RC | 77.40±0.18 | 43.39±1.68 | 25.44±0.44 |
| 10 | PIC20SC | 80.00±0.07 | 71.85±0.62 | 6.97±0.97 |
| 11 | PIC20SY | 79.86±0.08 | 66.89±0.92 | 4.13±1.92 |
| 12 | PIC23R | 76.75±0.11 | 72.01±0.09 | 26.39±1.30 |
| 13 | PIC22IF | 81.62±0.13 | 62.02±0.18 | 3.91±0.62 |
| 14 | PIC22RI | 78.53±0.05 | 60.02±0.35 | 21.61±0.95 |
| 15 | PIM8CR | 83.19±0.05 | 79.39±0.39 | 3.20±2.07 |
| 16 | PIC5CR | 81.45±0.08 | 80.82±0.97 | 14.97±3.08 |
| 17 | PIM9FI | 78.86±0.08 | 80.18±1.63 | 3.59±0.21 |
| 18 | PIM9CR | 86.14±0.03 | 80.63±0.59 | 3.51±0.87 |
| 19 | PIB9SR | 24.75±0.44 | 64.16±0.90 | 22.46±2.66 |
| 20 | PIB10CR | 73.19±0.96 | 87.64±0.74 | 21.84±1.13 |
| 21 | PIB10MR | 76.44±0.75 | 74.31±0.28 | 25.80±0.26 |
| 22 | PIB9MR | 77.52±0.42 | 78.02±0.92 | 7.75±0.70 |
| 23 | PIB9CR | 64.92±0.33 | 83.35±1.01 | 14.39±0.05 |

**S3 Table. Antioxidant activity of selected 23 isolates**

**All experiments were performed in triplicates; Data represented as Mean±SD**
