# Supplementary material for: Formulation and in-vitro functional evaluation of a Bacillus-based multi-strain probiotic consortium relevant to protein-energy malnutrition
Source: PLoS One. 2026 Mar 24;21(3):e0345821. doi: 10.1371/journal.pone.0345821 (PMC13012502; doi:10.1371/journal.pone.0345821)
Supplement: Attachment — Submitted filename: review PONE-D-25-60367.pdf [file pone.0345821.s004.pdf]

Manuscript Title:

Formulation and functional evaluation of multi-strain probiotic consortium as a potential therapeutic approach against protein-energy malnutrition
Manuscript Number: PONE-D-25-60367

Summary and overall impression:

This manuscript presents an *in vitro* evaluation of a multi-strain probiotic consortium (MSPC) composed of *Bacillus spizizenii*, *Bacillus tequilensis*, and *Bacillus rugosus*, selected from 23 isolates. The authors assess antagonistic interactions, proteolytic activity, anti-inflammatory activity, antioxidant activity (ABTS, DPPH, FRAP), synergistic growth patterns, and general microbial kinetics. They propose that the MSPC may serve as a potential therapeutic supplement for managing protein-energy malnutrition (PEM).

The topic is important, timely, and relevant, especially because PEM is a major public health issue. The manuscript contains well-organized experimental data and a logical flow from selection to consortium formulation and functional evaluation. However, major issues remain regarding statistical data, data interpretation, and details of figures.

Major issues:

Given that the study is conducted entirely *in vitro*, could the authors clarify whether such assays are sufficient to support the use of the term "potential therapeutic approach"?

The authors include the sentence: *"This study focuses on formulating and evaluating a multi-strain probiotic consortium (MSPC) as a potential therapeutic for PEM."* However, this statement only describes what was done and does not clearly express a specific research question or hypothesis. The authors should add a more precise objective or hypothesis.

In the Statistical Analysis section, the authors state that the experiments "…were performed in triplicate", but it is unclear whether this refers to technical replicates or biological replicates. This information is helpful for the reproducibility of the findings.

Figures 2–5 contain multiple critical issues. Figure 2B displays negative enzyme concentrations, which are not physically meaningful. The error bars are unclear. The figures use inconsistent font sizes; the authors should adjust the figures so that all labeling uses a consistent font size. The horizontal grid lines should be removed unless they serve a clear purpose.

The results are reported with three decimal places throughout the figures and text. Could the authors clarify whether this level of precision reflects the actual accuracy of the assays and instruments used? (e.g, 0.528±0.03)

The manuscript includes a brief limitation statement ("Further research is needed…", "Future clinical trials are warranted…"). Still, this language is quite general and does not specifically address the major methodological constraints of the study.

The authors may consider strengthening the Discussion by providing a deeper interpretation of the findings rather than primarily restating the results.

The conclusion would benefit from revision, because the current conclusion mainly restates earlier content; it does not provide a meaningful synthesis or deeper reflection on the implications and constraints of the study.

The manuscript provides some background on probiotics and PEM; however, I still find the rationale for selecting a *Bacillus*-based consortium somewhat limited. Would the authors consider expanding the justification for why these specific strains are suitable candidates for PEM intervention?

Minor issues:

Since the probiotic consortium consists of three Bacillus species, it may be helpful to specify "Bacillus-based multi-strain probiotic consortium" to improve clarity.

"Formulation and functional evaluation of multi-strain probiotic consortium" should be "a multi-strain probiotic consortium." This may cause readability issues.

Introducing the rationale for the MSPC earlier in the introduction could improve clarity. Bringing this information forward would create a smoother transition from the general discussion of malnutrition and probiotics to the specific focus of the study.

In the introduction section, please replace the "&" symbol in the phrase "small peptides & amino acids" with "and".

In the introduction, the sentence beginning with "Over the past decade, MSPC have shown…" uses the abbreviation MSPC as a plural noun. Using "MSPC has shown…" would be more grammatically appropriate.

Space Inconsistencies in Percentages. In the introduction, "According to a report by the World Health Organization (WHO)…" contains spacing issues.

A careful double-check of the references would help ensure consistent formatting throughout the manuscript.
