# Supplementary material for: Formulation and in-vitro functional evaluation of a Bacillus-based multi-strain probiotic consortium relevant to protein-energy malnutrition
Source: PLoS One. 2026 Mar 24;21(3):e0345821. doi: 10.1371/journal.pone.0345821 (PMC13012502; doi:10.1371/journal.pone.0345821)
Supplement: Attachment — Submitted filename: Response to Reviewers.docx [file pone.0345821.s006.docx]

**Major Comments**

1. **Given that the study is conducted entirely in vitro, could the authors clarify whether such assays are sufficient to support the use of the term “potential therapeutic approach”?**

**Response to reviewer:** We thank the reviewer for this important point. We agree that in vitro assays alone cannot establish therapeutic efficacy. In the revised manuscript, we have clarified that the term “potential therapeutic approach” is used to indicate preliminary functional evidence rather than clinical or in vivo validation. We have revised the Abstract, Discussion, and Conclusion to explicitly state that the findings are based on in vitro assays and that in vivo and clinical studies are required to confirm therapeutic applicability.

2. **The authors include the sentence: “This study focuses on formulating and evaluating a multi-strain probiotic consortium (MSPC) as a potential therapeutic for PEM.” However, this statement only describes what was done and does not clearly express a specific research question or hypothesis. The authors should add a more precise objective or hypothesis.**

**Response to Reviewer**: We appreciate this suggestion. The objective of the study has now been revised to clearly articulate the research question and rationale. A concise and specific objective statement has been added to the Introduction.

3. **In the Statistical Analysis section, the authors state that the experiments “…were performed in triplicate”, but it is unclear whether this refers to technical replicates or biological replicates. This information is helpful for the reproducibility of the findings.**

**Response to Reviewer:** We thank the reviewer for highlighting this point. The manuscript has been revised to clarify that all experiments were conducted using technical triplicates, unless otherwise specified.

4. **Figures 2–5 contain multiple critical issues. Figure 2B displays negative enzyme concentrations, which are not physically meaningful. The error bars are unclear. The figures use inconsistent font sizes; the authors should adjust the figures so that all labeling uses a consistent font size. The horizontal grid lines should be removed unless they serve a clear purpose.**

**Response to Reviewer:** We appreciate the detailed feedback. All figures have been carefully revised. Negative values have been corrected, error bars are now clearly defined in the figure legends, font sizes have been standardized across all figures, and unnecessary horizontal grid lines have been removed.

5. **The results are reported with three decimal places throughout the figures and text. Could the authors clarify whether this level of precision reflects the actual accuracy of the assays and instruments used? (e.g, 0.528±0.03)**

**Response to Reviewer:** We agree with the reviewer that excessive decimal precision may imply an unrealistic level of accuracy. The manuscript and figures have been revised to limit numerical values to two decimal places, consistent with assay sensitivity.

6. The manuscript includes a brief limitation statement (“Further research is needed…”, “Future clinical trials are warranted…”). Still, this language is quite general and does not specifically address the major methodological constraints of the study.

**Response to Reviewer:** We thank the reviewer for this constructive and valuable suggestion. We agree that the limitations should be more clearly articulated. The manuscript has been revised to explicitly state that the present work represents the first phase of a multi-stage research framework. This initial phase focuses on in vitro functional screening and consortium formulation, while subsequent phases are planned to include in vivo animal studies and metagenomic analyses to validate host–microbiome interactions and functional outcomes. These clarifications have been incorporated into the revised manuscript.

7. **The authors may consider strengthening the Discussion by providing a deeper interpretation of the findings rather than primarily restating the results.**

**Response to Reviewer:** We thank the reviewer for this insightful comment. We agree that the original Discussion was primarily descriptive. In the revised manuscript, the Discussion has been substantially rewritten to emphasize interpretation of the findings by explaining their biological relevance to protein-energy malnutrition, comparing the results with previously published studies, and highlighting how the observed functional properties of the consortium may contribute to nutritional support in PEM rather than restating numerical outcomes.

8. **The conclusion would benefit from revision, because the current conclusion mainly restates earlier content; it does not provide a meaningful synthesis or deeper reflection on the implications and constraints of the study.**

**Response to Reviewer:** The Conclusion has been revised to provide a concise synthesis of key findings, their implications, and the constraints of the study, rather than reiterating experimental details.

9. **The manuscript provides some background on probiotics and PEM; however, I still find the rationale for selecting a Bacillus-based consortium somewhat limited. Would the authors consider expanding the justification for why these specific strains are suitable candidates for PEM intervention?**

**Response to Reviewer:** We thank the reviewer for this valuable suggestion. The Introduction and Discussion have been expanded to better justify the selection of Bacillus species, emphasizing their spore-forming ability, environmental resilience, enzymatic activity, and stability under storage and gastrointestinal conditions.

**Minor comments**

1. **Since the probiotic consortium consists of three Bacillus species, it may be helpful to specify "Bacillus-based multi-strain probiotic consortium" to improve clarity.**

**Response to Reviewer:** We thank the reviewer for this helpful suggestion. To improve clarity and precision, we have revised the manuscript to consistently refer to the formulation as a “Bacillus-based multi-strain probiotic consortium” at appropriate locations.

2. **“Formulation and functional evaluation of multi-strain probiotic consortium” should be “a multi-strain probiotic consortium.” This may cause readability issues.**

**Response to Reviewer:** We thank the reviewer for pointing out this grammatical issue. The title and corresponding text have been revised to improve readability and grammatical accuracy.

3. **Introducing the rationale for the MSPC earlier in the introduction could improve clarity. Bringing this information forward would create a smoother transition from the general discussion of malnutrition and probiotics to the specific focus of the study.**

**Response to Reviewer:** We appreciate this suggestion. The Introduction has been revised to introduce the rationale for the multi-strain probiotic consortium earlier, thereby improving the logical flow from general background to study-specific focus.

4. **In the introduction section, please replace the “&” symbol in the phrase “small peptides & amino acids” with “and”.**

**Response to Reviewer:** We thank the reviewer for this observation. The ampersand symbol has been replaced with “and” to maintain formal scientific writing style.

5. **In the introduction, the sentence beginning with “Over the past decade, MSPC have shown…” uses the abbreviation MSPC as a plural noun. Using “MSPC has shown…” would be more grammatically appropriate.**

**Response to Reviewer:** We appreciate the reviewer’s attention to grammatical accuracy. The sentence has been revised to ensure correct singular usage of the abbreviation.

6. **Space Inconsistencies in Percentages. In the introduction, “According to a report by the World Health Organization (WHO)…” contains spacing issues.**

**Response to Reviewer:** We thank the reviewer for noting this formatting issue. Spacing inconsistencies in percentage values have been corrected throughout the manuscript to ensure uniform formatting.

7. **A careful double-check of the references would help ensure consistent formatting throughout the manuscript.**

**Response to Reviewer:** We appreciate this comment. The reference list has been carefully reviewed and reformatted to ensure consistency in journal abbreviations, author formatting, and DOI presentation, in accordance with journal guidelines.

**Comments of portal**

1. **In the introduction section, when talking about malnutrition in India, supporting statistics needs to be added. Without any data, the claim in the study feels vague.**

**Response to Reviewer:** We thank the reviewer for this important observation. We agree that quantitative data are essential to strengthen the context of malnutrition in India. Accordingly, the Introduction has been revised to include recent and authoritative national and global statistics highlighting the prevalence and burden of malnutrition in India.

2**. In the introduction section, in the 2nd paragraph, it has been reported that several probiotic strains have been shown to enhance digestive enzyme activity. Which strains have these properties? This needs to be further explained.**

**Response to Reviewer:** We appreciate the reviewer’s request for clarification. The Introduction has been revised to explicitly identify probiotic genera and representative strains reported to enhance digestive enzyme activity, thereby improving clarity and scientific grounding.

**3. In the Materials and Methods section, proteolytic activity and anti-inflammatory methods, formula that has been used for the calculation implies that concentration equals intercept divided by absorbance. In colorimetric assays, absorbance usually increases with concentration. Your formula suggests an inverse relationship. You must verify this against your standard curve data. A standard curve typically follows the linear equation y = mx+c.**

**Response to Reviewer:** We thank the reviewer for carefully identifying this methodological inconsistency. We have re-verified the standard curve data and corrected the calculation method to reflect the appropriate linear relationship between absorbance and concentration based on the equation y = mx + c.

**4. The statistical analysis reported for the analysis of the data is the calculation of the mean and standard deviation. This is not enough, and since different groups are being compared, statistical analysis such as One-Way ANOVA and Post-hoc Test need to be used. To confirm, for instance, strain A is superior to strain B, without the analysis of variance and a p-value, this is not acceptable and it requires statistical support.**

**Response to Reviewer:** We thank the reviewer for this important comment. The primary objective of the present study was functional screening and characterization of individual strains and the formulated consortium, rather than hypothesis-driven comparison between experimental treatment groups. Each strain and the MSPC were evaluated independently under identical assay conditions, and results were therefore summarized using descriptive statistics (mean ± standard deviation). For this, we have reframed our statements in result and discussion. We have removed words like significantly higher, statistically superior, and significant difference.

Since no experimental grouping or treatment-based comparison was performed, inferential statistical tests such as one-way ANOVA were not applied. This approach is consistent with exploratory in vitro screening studies aimed at identifying functional potential rather than establishing statistical superiority between strains. We have clarified this rationale in the Statistical Analysis section of the manuscript.

**5. In the results section, figure captions need to have a detailed explanation of what the figure is showing, including what each axis represents.**

**Response to Reviewer:** We thank the reviewer for this suggestion. All figure captions have been revised to clearly describe the experimental context, axes, units, and comparisons shown. Also, as per comment no. 8 below, the respective figure has been removed, and the table has been kept.

**6. In the results section, the antagonistic activity section, PIB12RB strain, has been written twice.**

**Response to Reviewer:** We thank the reviewer for pointing out this oversight. The duplication of the PIB12RB strain in the antagonistic activity section has been corrected.

**7. In the results, proteolytic activity, why was the value of 0.365 selected? Also, in the same section, it has been reported that your consortium is less effective than the single strain of PIG5CI, with the value of 0.615 mg/mL. In the introduction and discussion, it has been claimed that the MSPC offers "synergistic effects." Scientifically, synergy implies that the combination is greater than the sum (or at least the best) of its parts. Here, your consortium is less effective than the single-strain PIG5CI. This needs to be further explained. In addition, mg/mL is not a common unit for the proteolytic activity as the activity products (tyrosine) have been measured and the no actual physical mass of the enzyme. The common unit is Units/mL, and this needs to be reviewed.**

**Response to Reviewer:** We thank the reviewer for this insightful comment. The selection of the proteolytic activity threshold and the interpretation of synergistic effects have been clarified, and the unit of proteolytic activity has been corrected throughout the manuscript.

The value of PIG3IR (**0.365 Units/mL)** was not selected arbitrarily. This strain was prioritized because, in addition to exhibiting moderate proteolytic activity, it demonstrated **superior anti-inflammatory activity** compared to other isolates. Given the multifactorial nature of protein-energy malnutrition, strain selection for consortium development was based on **overall functional performance**, rather than maximal activity in a single assay.

Furthermore, the interpretation of “synergy” has been revised to reflect **functional complementarity** rather than numerical enhancement of a single parameter. Although the MSPC exhibited lower proteolytic activity than the single strain PIG5CI, the consortium was designed to integrate complementary enzymatic, antioxidant, and anti-inflammatory properties, along with improved strain compatibility, to achieve broader functional relevance.

Finally, proteolytic activity units have been corrected from mg/mL to **Units/mL.**

**8. Table 1 and Figure 2A, Table 2 and Figure 3, present the same data, except for the MSPC value. This is redundant, and one should be selected to report the data.**

**Response to Reviewer:** We agree with the reviewer that presenting identical datasets in both tabular and graphical formats is redundant. The data presentation has been streamlined by retaining only one format.

**9. In Figure 4, the data points jump from 7 hours to 24 hours. The actual growth curve has been missed and therefore, growth kinetics cannot be compared and discussed.**

**Response to Reviewer:** We thank the reviewer for raising this point. The growth curve analysis was primarily designed to monitor early growth behavior up to 7 h. However, additional measurements were performed at 24 h and 48 h to confirm entry into and maintenance of the stationary phase. Intermediate time points between 7 h and 24 h were therefore not included, which limited detailed kinetic comparisons during the exponential phase. This limitation has now been explicitly acknowledged in the revised Discussion.

**10. In the discussion section, the reported activity is 200 times lower than the literature you cited. Yet, it has been concluded: "Our results align with previous findings." This requires clarification and revision.**

**Response to Reviewer:** We thank the reviewer for highlighting this important discrepancy. The Discussion has been revised to avoid overstating alignment with previous studies and to contextualize the lower magnitude of activity in terms of strain specificity and assay conditions.
