# Supplementary material for: Formulation and in-vitro functional evaluation of a Bacillus-based multi-strain probiotic consortium relevant to protein-energy malnutrition
Source: PLoS One. 2026 Mar 24;21(3):e0345821. doi: 10.1371/journal.pone.0345821 (PMC13012502; doi:10.1371/journal.pone.0345821)
Supplement: Attachment — Submitted filename: Response_to_Reviewers_auresp_2.docx [file pone.0345821.s007.docx]

**Response to Reviewers**

**Manuscript ID:** PONE-D-25-60367R1

**Title:** Formulation and in vitro functional evaluation of a Bacillus-based multi-strain probiotic consortium relevant to protein-energy malnutrition

Dear Editor and Reviewers,

We sincerely thank the Academic Editor and the reviewers for their careful evaluation of our manuscript and for the constructive comments provided during the review process. We appreciate the positive feedback and are grateful for the opportunity to revise our manuscript.

We are pleased to note that the reviewers acknowledged that the comments from the previous round have been adequately addressed and that the revisions improved the clarity and scientific presentation of the manuscript. Following the reviewer’s suggestions, we have further revised the manuscript to refine the interpretation of the findings, improve the overall flow of the text, and enhance the clarity of error bar presentation in the figures.

All changes made in the revised manuscript are **clearly highlighted** in the marked-up version submitted as **“Revised Manuscript with Track Changes.”** An unmarked, clean version of the manuscript has also been provided as requested.

Below, we provide our point-by-point response to the reviewer’s comment.

**Reviewer - 1**

**Comment:** The authors have adequately addressed the comments raised in the previous round of review. The revisions have improved the clarity and scientific presentation of the manuscript. However, further refinement in the interpretation of the findings, overall flow of writing, and clearer presentation of error bars in the figures would strengthen the manuscript.

**Response:**
We sincerely thank the reviewer for the positive assessment of our revised manuscript and for the valuable suggestions to further improve its quality.

**Refinement of the interpretation of findings**

The discussion section has been carefully revised to provide a clearer and more precise interpretation of the experimental findings. Additional explanations have been incorporated to better relate the results to the functional characteristics of the Bacillus-based probiotic consortium and its potential relevance to protein-energy malnutrition.

**Improvement in the overall flow of writing**

The manuscript text has been edited throughout to improve readability and logical flow. Sentences and paragraph transitions in the Results and Discussion sections have been refined to ensure clearer progression of ideas.

**Clearer presentation of error bars in figures**
The figures have been revised to improve the visibility and clarity of the error bars. The figure legends have also been updated to explicitly state the statistical parameters represented by the error bars (mean ± standard deviation).

We believe these revisions have further improved the clarity, presentation, and scientific interpretation of the manuscript.

Once again, we sincerely thank the editor and reviewer for their constructive feedback and for considering our revised manuscript. We hope that the changes made satisfactorily address the reviewer’s concerns and that the manuscript is now suitable for publication.

Sincerely,
**Priya Mori**
(On behalf of all authors)
